# Radiological Patterns of Lung Injury on HRCT in COVID-19 Patients: An Experience From Tertiary Care Centre in India

**DOI:** 10.7759/cureus.48479

**Published:** 2023-11-07

**Authors:** Amit K Verma, Siddharth Mishra, Nitin A Dikshit, Anil Rawat, Saurabh Kumar

**Affiliations:** 1 Radiodiagnosis, King George's Medical University, Lucknow, IND

**Keywords:** ground-glass opacities, pneumonia, ct, covid-19, coronavirus disease 2019

## Abstract

Introduction: During the outbreak of the COVID-19 pandemic, radiological examinations became crucial in assessing the severity and progression of lung injuries in COVID-19 patients.

Aim: This study was done to identify radiological patterns of lung injuries in COVID-19 patients, assess lobar involvement, and perform CT severity scoring on symptomatic patients on a baseline scan.

Methods and material: All the RT-PCR-positive patients were retrospectively enrolled in the study from August 16, 2020 to October 12, 2020. The final heart-resolution computed tomographic (HRCT) thorax data of a total of 119 COVID-19 patients was analyzed using simple statistical methods. A p-value of less than 0.05 (p < 0.05) was considered statistically significant.

Results: A total of 119 HRCT thorax scans of symptomatic indoor RT-PCR-positive patients were reviewed. Over 50% of the patients were under 50 (n = 66; 25 = 5; 25-50 = 61). Males > females: 3.25:1 (M = 91; F = 28). Peripheral involvement dominated (n = 90). Both lungs were affected equally, but the right lower lobe was more involved (n = 103) than the left (n = 98). Inpatient care was needed for 64.70% of CT severity score (CTSS) 10 COVID-19 patients (n = 77). Most positive CT scans (n = 115) revealed ground glass opacities (n = 112; 97.39%). Vascular dilatation or vasculitis (n = 65; 56.62%) and organizing pneumonia-like changes (n = 61; 53.04%) were also common. Vascular enlargement (56.53 percent, n = 65) and reverse halo (52.17%, n = 60) were the most common CT signs.

Conclusion: The most common chest CT finding in COVID-19 was ground-glass opacity in peripheral distribution with extensive lung involvement. These ground glass opacities may coalesce into consolidations. Vascular dilatation, organizing pneumonia, interlobular septal thickening, and crazy paving were other important imaging characteristics.

## Introduction

An outbreak of unexplained pneumonia was reported in December 2019 in Wuhan, in the Hubei province of China [1,2]. The causative agent was identified as a novel coronavirus. On February 11, 2020, the 2019 novel coronavirus was named severe acute respiratory syndrome coronavirus 2 (SARS-CoV-2) by the International Committee on Taxonomy of Viruses (ICTV), and the disease was named the coronavirus disease (COVID-19) by the World Health Organization (WHO) [3,4]. Since then, COVID-19 has spread to several parts of the world and was declared a pandemic by the WHO on March 11, 2020.

Human-to-human transmission of the virus occurs via droplets and fomites [5]. The disease presents with a dry cough and fever, and nonspecific symptoms include headache, dyspnea, fatigue, and muscle soreness [6]. Severe cases account for approximately 20%, and mortality is approximately 3% [7].

For screening purposes, a swab from the upper respiratory tract, including the naso and oropharynx, is used for antigen-based testing; however, COVID-19 was confirmed by gold standard reverse transcription-polymerase chain reaction (RT-PCR). However, lung changes may be seen on chest CT imaging earlier than the RT-PCR. High-resolution computed tomographic (HRCT) chest imaging has been used as a tool for prompt diagnosis and assessment of disease severity in patients with symptoms of COVID-19.

Typical CXR findings of COVID-19 include bilateral peripheral and basal multifocal airspace opacities (ground-glass opacity (GGO) and consolidation). Various patterns of CXR findings may be observed among the local COVID-19-positive patients. This study was conducted with the objective of determining the chest CT findings of RT-PCR-positive COVID-19 patients admitted to a tertiary care hospital in North India.

## Materials and methods

Study design and sampling

This is a retrospective review and cross-sectional study of prospectively collected data. After approval from the institutional ethics committee, the study was conducted in the Department of Radiodiagnosis. To maintain the privacy, integrity, and quality of the data, only admitted patients were included. Per WHO guidelines, patients with positive RT-PCR assays were considered confirmed cases of COVID-19 [8]. All the symptomatic RT-PCR-positive COVID-19 patients admitted to the Infectious Disease Hospital (IDH) who had a baseline CT thorax done on the department CT scanner were included in the study. Informed verbal or written consent for imaging, data collection, and publication of the data was obtained from all patients or their legal guardians at the time of scanning.

Inclusion Criteria of Patients 

All known COVID-19 patients with respiratory symptoms were admitted to the IDH who underwent a CT thorax in the department.

Exclusion Criteria of Patients

1. Pediatric patients with mild symptoms and age <5 years, considering the risk of radiation.

2. Known cases of diffuse lung diseases such as ILD, diffuse pulmonary tuberculosis, occupational lung diseases, and previously collapsed or fibrosis lungs.

3. Patients who refused to consent to the collection or publication of the data.

CT thorax-imaging protocol

CT scans of all the included patients were done in the Department of Radiodiagnosis on a 128-slice multidetector CT scanner (Philips Brilliance). HRCT scans of the chest were performed using the institutional protocol without the use of intravenous contrast. The images were acquired under helical CT, with the region of interest extending from the lower neck to the upper abdomen. Single-breath-hold scans were acquired whenever possible to avoid respiratory motion artifacts. The raw images were subsequently reconstructed into 0.625-mm-thin sections without intervening gaps. Thin MIP, MinIP, coronal, and sagittal images were reconstructed per the requirements.

Data collection and imaging interpretation

The CT imaging raw data and reconstructed images of the study group were retrieved from the digital data bank. All the scans were reviewed in the light of clinical knowledge by two staff radiologists with more than five years of experience. Lung involvement was graded under different variables concerning anatomical involvement (e.g., lobes and segments), types of imaging findings (e.g., ground glass and consolidations), distribution gradients (e.g., peripheral, central, or diffuse), and other associated findings (e.g., pleural or pericardial effusions and lymphadenopathy). The severity of the findings was graded per the CT severity score (CTSS). For calculation of the CTSS, each lung was divided into 10 segments corresponding to 10 bronchopulmonary segments of the right lung and eight segments of the left lung. The apico-posterior segment of the left lung was further divided into apical and posterior segments, and similarly, the anteromedial basal segment was further divided into anterior and basal segments. The radiological involvement of each segment was scored from 0 to 2 where 0 corresponded to no involvement, 1 to the involvement of <50%, and 2 to the involvement of >50% of the segment. The sum of each segment from both the lobes was given as the total score with a maximum CTSS of 40.

Patterns of radiological findings

The radiological pattern of lung injury was documented in terms of radiological findings. These patterns included GGOs, consolidations, parenchymal bands, bronchial wall thickening, organizing pneumonia patterns, vascular dilatation, and crazy paving. Extra-parenchymal findings such as pleural thickening, pleural effusion, pericardial effusions, and mediastinal lymphadenopathy were also noted. Some of the important CT signs (air bronchogram, target sign, reverse halo sign, etc.) were also documented to assess their prevalence among the findings. Patterns of distribution were divided into peripheral, central, diffuse, or random. Involvement of the individual lobe or segment of the lobe was also assessed.

Data management and statistical analysis

Extensive efforts were made to ensure the quality of the data. The final data of a total of 119 COVID-19 patients who had CT chests were analyzed using simple statistical methods. Quantitative data of demographics as well as findings were expressed in terms of maximum, minimum, mean, and standard deviation. Similarly, qualitative data was expressed in a proportion or percentage. A p-value of less than 0.05 (p < 0.05) was considered statistically significant.

## Results

Total 119, CT chest scans of RT-PCR assay-positive, symptomatic indoor patients were reviewed. Their demographic and imaging data were stored and assessed to interpret the findings as per the objective of the study. Age distribution was very inhomogeneous. Patients from five to 81 years of age (mean age: 48.47 years) were affected by the disease and needed hospital admissions. More than half of our admitted patients were below 50 years or below (n=66; less than or equal to 25 years=5; 25-50 years=61); however, 53 admitted patients were above 50 years of age. Commonly compared to females, the male population was affected more commonly with a male-to-female ratio of 3.25:1 (M=91; F=28).

**Figure 1:**
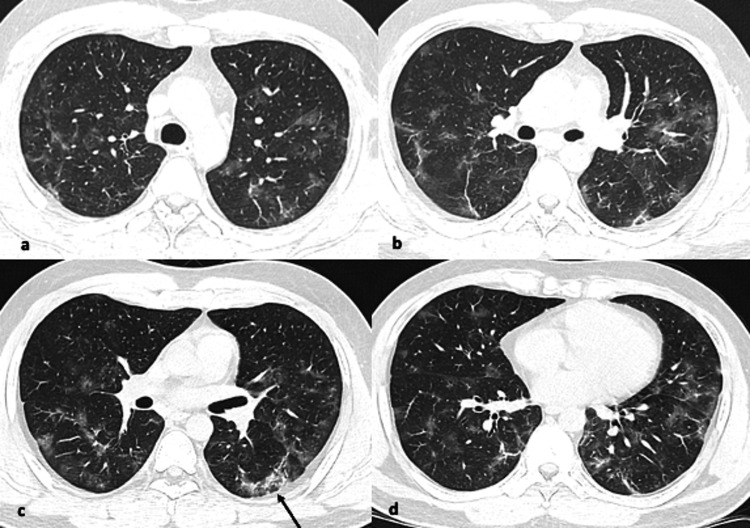
(a-d) Axial CT images from different known COVID-19 patients showing pattern of findings (arrows). Images showing patchy ground glass opacities scattered throughout bilateral lungs, slightly denser in left posterior subpleural lower lobe with reverse halo sign (arrow).

**Figure 2:**
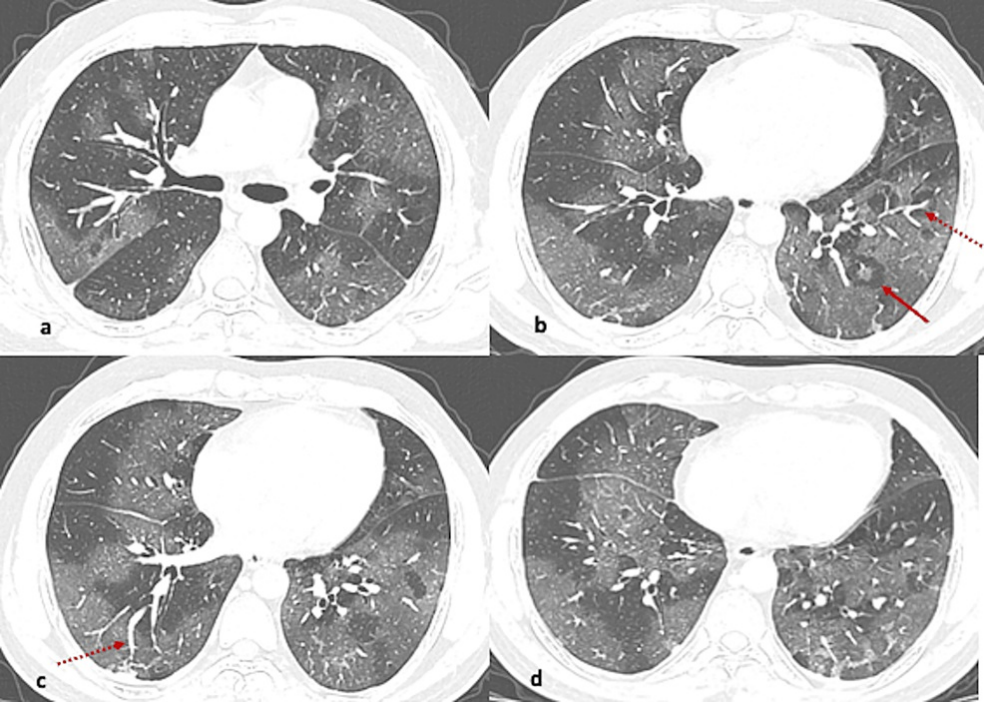
(a-d) Axial CT images from different known COVID-19 patients showing pattern of findings (arrows). Extensive ground glass opacities diffusely involving bilateral lungs giving a mosaic appearance. There is halo with target sign (arrow) and vascular dilatation (dotted arrow). Findings resemble subacute hypersensitivity pneumonitis.

**Figure 3:**
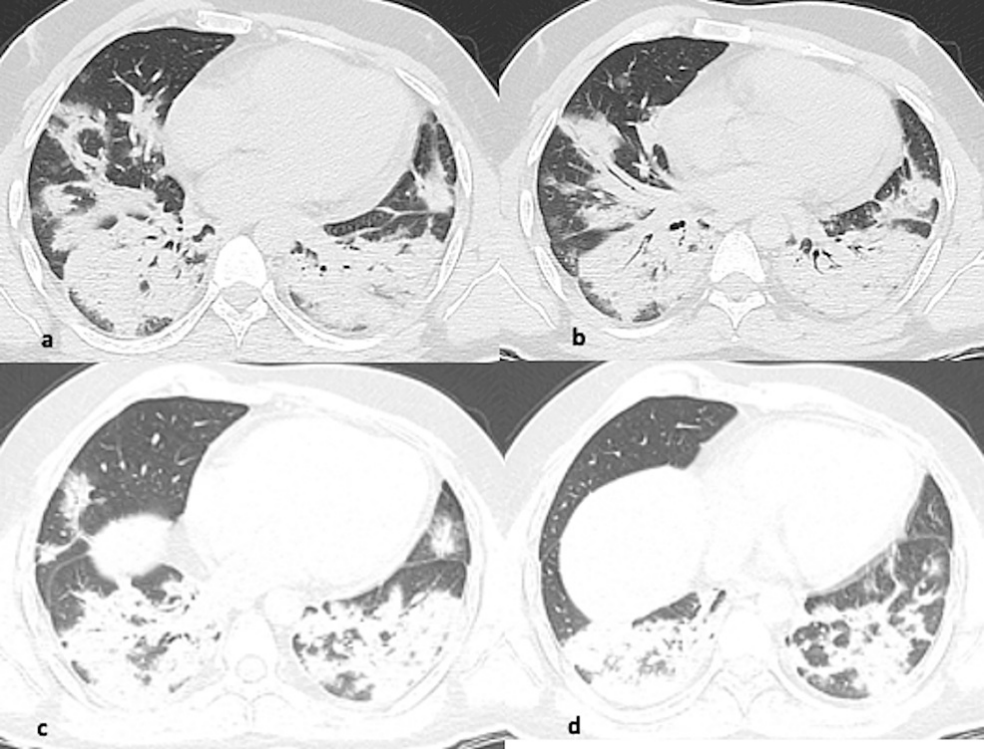
(a-d) Axial CT images from different known COVID-19 patients showing pattern of findings (arrows). Confluent airspace consolidation with air-bronchogram in both the lungs, is more predominant in lower lobes and peribronchial distribution. Areas of reverse halo sign are also present. The appearance resembles diffuse alveolar edema, hemorrhage, or organizing pneumonia.

Out of 119, total 115 patients have lung parenchymal involvement on CT images while the remaining four patients have normal CT chest without concerning imaging findings. In terms of distribution, peripheral involvement was most common (n=90) (Figures 1a-1d). Diffuse lung involvement (Figures 2a-2d) was the second most common pattern (n=14) followed by random opacities (n=10) however, only one patient has entirely central distribution (n=1) (Figures 3a-3d).

There was no significant difference in laterality of disease. The right and left lungs were affected in nearly similar numbers of patients (nr= 106, nl =106). There was a caudal to cranial gradient in each lung with lower lobes affected most commonly. Right lower lobe involvement was most common (n=103) followed by involvement left lower lobe (n=98). The left upper lobe was third most commonly involved lobe (n=91) due to its larger area compared to the right side. Right middle and upper lobes were involved in 89 and 84 patients, respectively. Lung changes of COVID-19 also have a posterior to anterior gradient which mean posterior peripheral lungs were most commonly involved in all the lobes. The posterior basal (nr=99, nl =93) and superior segments (nr = 89, nl = 84) of the lower lobes were the most commonly involved in either of the lungs. A medial segment of the right middle lobe (n=11) was over all least commonly involved segment of the lungs while medial basal (n=53) and anterior basal (n=53) segments of the lower lobe were least commonly involved in the left lung (Tables 1, 2). The CTSS was calculated by summing up individual scores of each segment and lobe of both the lungs. Nearly two third of COVID-19 patients (n=77, 64.70%) who had a CTSS less than or equal to 10, required hospital admission in in-patient facility. Out of these, 42 (35.29%) patients had a CTSS of 10-19 while other 35 (29.41%) patients had a CTSS of less than or equal to 20.

**Table 1:** Segmental involvement of right lung

Lung involvement	no of cases
Right	106
Upper lobe	84
Superior segment	58
Anterior segment	62
Posterior segment	77
Middle lobe	89
Medial Segment	11
Lateral segment	63
Lower lobe	103
Superior Segment	89
Medial Basal segment	79
Lateral basal segment	70
Anterior Basal Segment	52
Posterior Basal segment	99

**Table 2:** Segmental involvement of left lung

Lung involvement	no of cases
Left	106
Upper lobe	91
Superior segment	65
Anterior segment	60
Posterior segment	81
Lingular lobe	
Superior lingula	56
Inferior Lingula	79
Lower lobe	98
Superior Segment	84
Medial Basal segment	53
Lateral basal segment	69
Anterior Basal Segment	53
Posterior Basal segment	93

**Figure 4:**
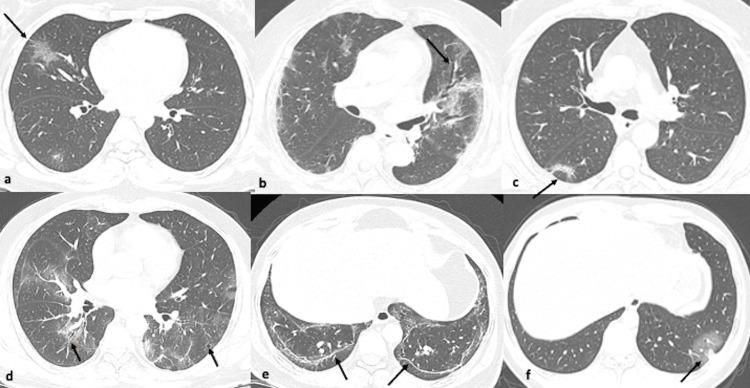
(a-f) Axial CT images from different known COVID-19 patients showing pattern of findings (arrows). Ground glass with crazy paving (a); ground glass with bronchial dilatation (b); reverse halo sign resembling organizing pneumonia (c), small cysts and bronchial dilatation (d), lower lobes parenchymal bands (e) and vascular dilatation and target/halo sign (f).

Among the positive CT scans (n=115), GGOs were the most common and were present in nearly all cases (n = 112; 97.39%) (Figures 1a-1d). Vascular dilatation or vasculitis changes (n=65; 56.62%) and organizing pneumonia like changes (n=61; 53.04%) were other common findings. Visually mild to moderate bronchial wall thickening was present in 51.30% (n=59), followed by parenchymal bands (n=58; 50.43%) and cylindrical bronchial dilatation/bronchiectasis (n=53; 46.08%) (Figures 4a-4f).

**Figure 5:**
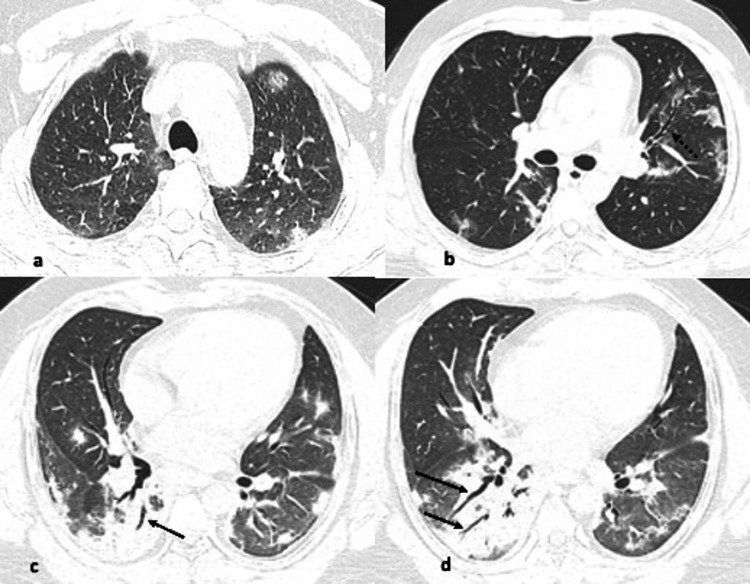
(a-d) Axial CT images from different known COVID-19 patients showing pattern of findings (arrows). Patchy lower lobe predominant ground glass opacities in the lungs. There is right lower lobe confluent infrahilar consolidation with bronchial dilatation and air bronchogram (arrow). Parahilar bronchial dilatation in lingula as well (dotted arrow).

In our study, acute consolidations were seen only 36% (n=41) of the patients (Figures 5a-5d), crazy paving (n=17; 14.78%) and some degree of septal thickening (n=16; 13.91%) were present in nearly equal number of patients.

**Figure 6:**
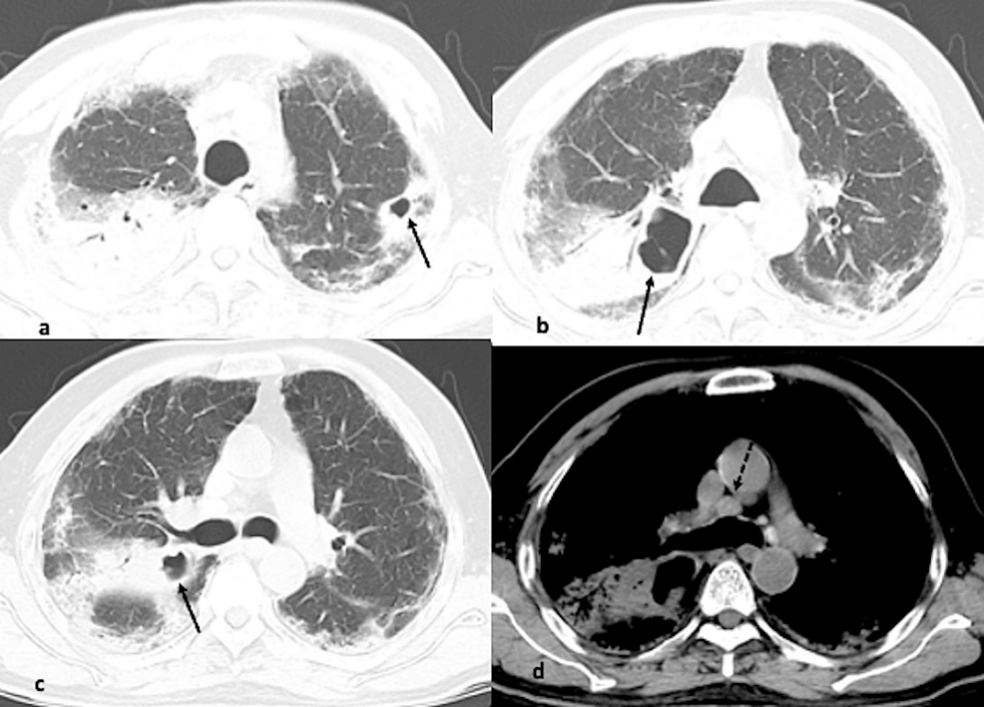
(a-d) Axial CT images from different known COVID-19 patients showing pattern of findings (arrows). Lung and mediastinal window showing patchy and confluent peripheral predominant consolidations in both the lungs with cavitary changes (arrow) in bilateral upper lobes. There are early organizing changes in subpleural areas. Few prominent lower paratracheal nodes (dotted arrow).

**Figure 7:**
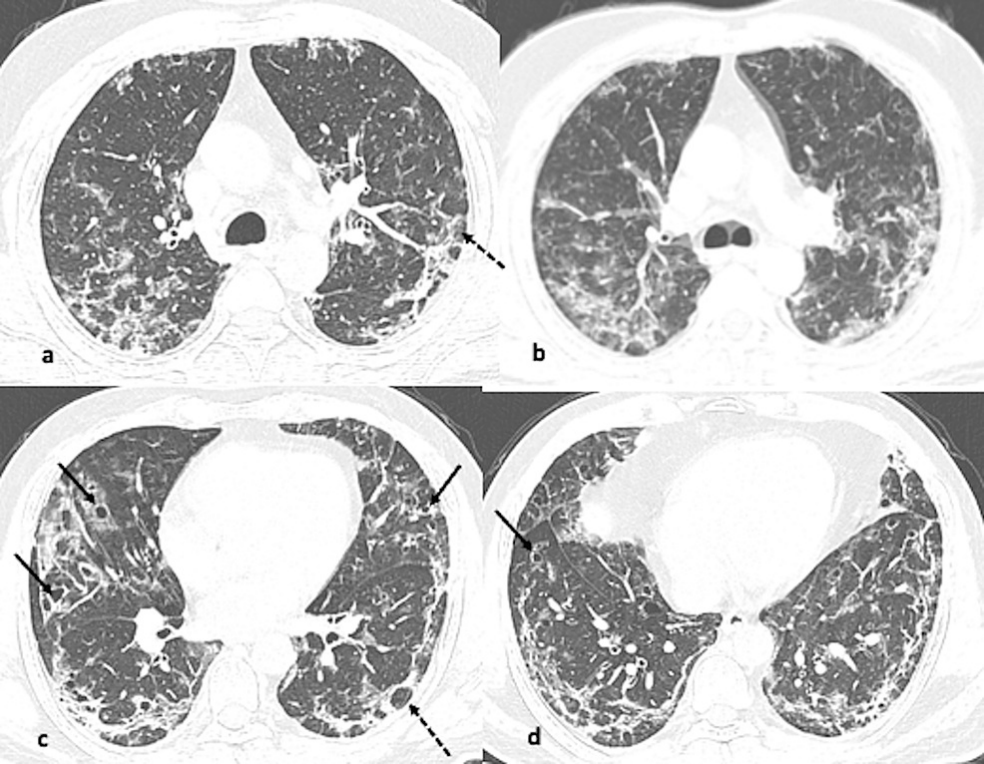
(a-d) Axial CT images show diffusely scattered ground glass changes in bilateral lungs, more predominant posteriorly. Multiple small cysts/pneumatoceles (arrow) and organizing pneumonia like changes/reverse halo signs (dotted arrow) are present in affected areas.

Cavitation changes and small cysts in the affected lungs were uncommon and seen in nearly 4.34% (n=5) and 1.73 % (n=2) of the patients, respectively (Figures 6a-6d, 7a-7d).

**Figure 8:**
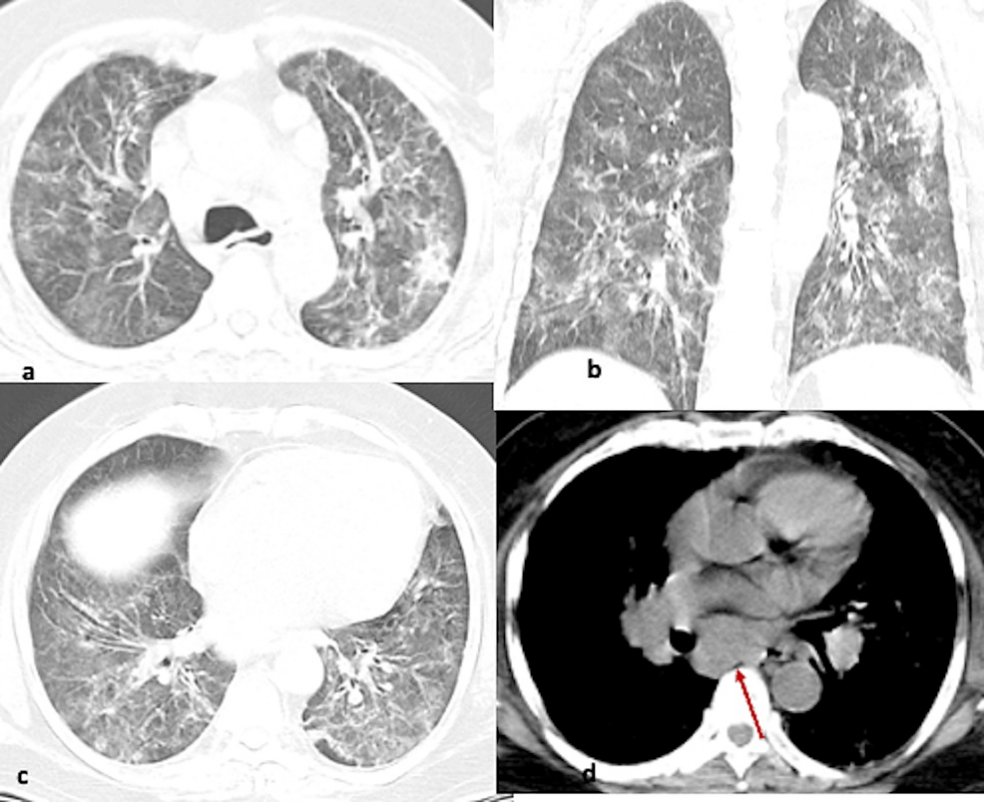
(a-d) Axial and coronal CT chest images in lung and mediastinal window. Extensively diffuse ground glass opacities throughout both the lungs. Small left upper lobe peripheral consolidation. There are significantly enlarged hilar and subcarinal lymph nodes (arrow) which resolved on 6months follow-up.

**Figure 9:**
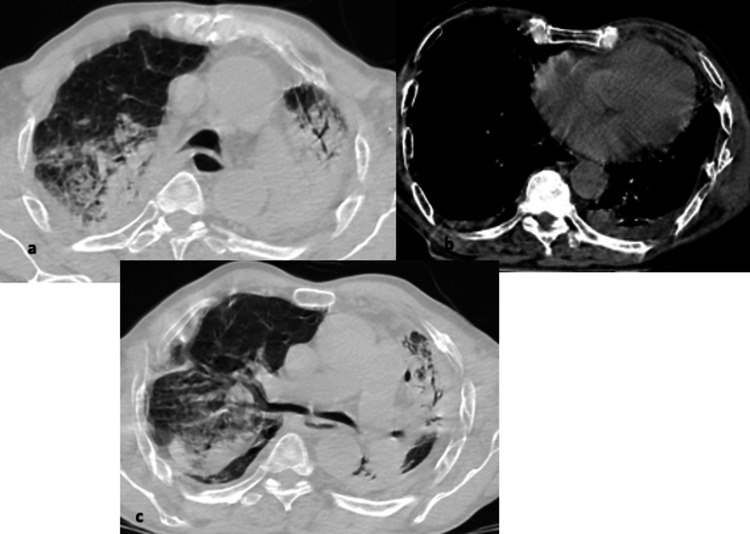
(a-c) Axial images from CT chest showing confluent consolidations bilaterally with small pleural effusions. Image quality affected by breathing and motion artifacts.

Mediastinal lymphadenopathy without additional cause was seen in three patients (2.60%) while another patient had lymphadenopathy secondary to known non-Hodgkin’s lymphoma (NHL) (Figures 8a-8d). Pleural effusion was seen in six patients (5.21%) (Figures 9a-9c) while one patient had pericardial effusion (0.86%) without pleural or pericardial thickening, respectively. The pericardial effusion and pneumothorax were least common findings in the study group with one patient of each (0.86%).

**Figure 10:**
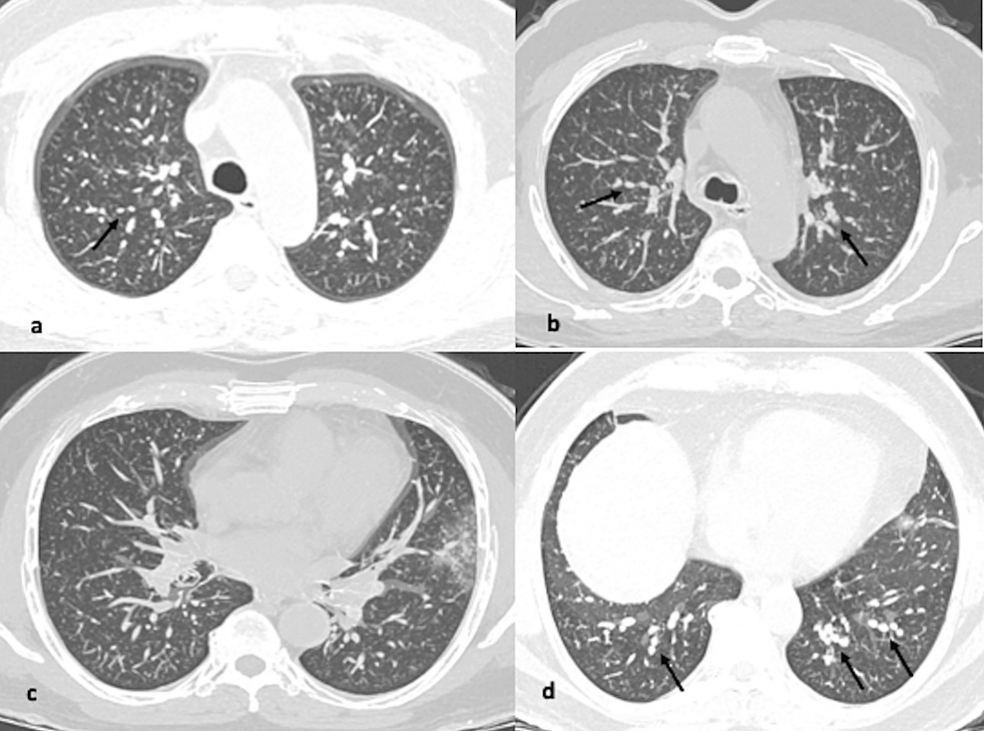
(a-d) Axial CT chest images showing mild peripheral ground glass in lingula. There are scattered micronodules in bilateral upper and lower lobes with vascular beading/vascular tree in bud appearance (arrows).

**Figure 11:**
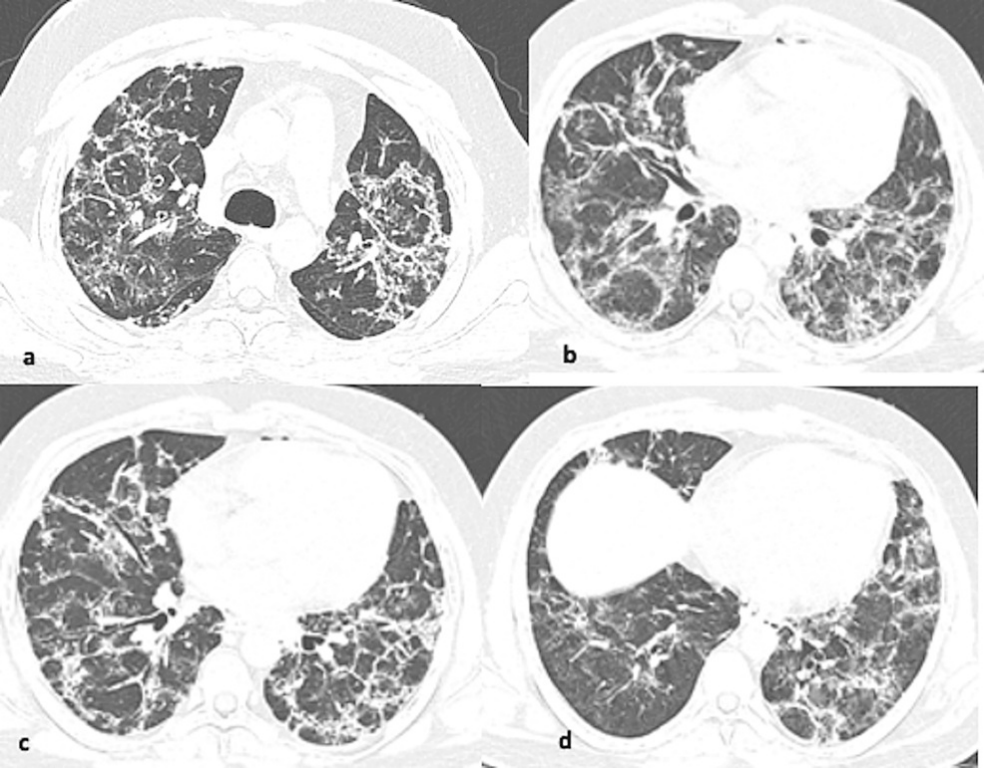
(a-d) Axial CT chest images showing extensive scattered small consolidations with ground glass opacities and interlobular septal thickening resembling organizing pneumonia pattern diffusely involving bilateral lungs. OP changes also include halo and reverse halo signs as well as mild bronchial dilatation.

Among few CT signs we assessed, vascular enlargement and reverse halo signs of were most common, contributing 56.53% (n=65) and 52.17% (n=60), respectively. Halo sign was seen in 20 patients (17.39%) followed by air-bronchogram sign in 19 patients (16.67%) and reticular pattern in 17 patients (14.78%) (Figures 10a-10d). Vascular beading (n=1; 0.86%), vascular tree-in-bud (n=2; 1.73%) and air trapping (n=1; 0.86%) were uncommon. Vascular enlargement, beading and vascular tree-in-bud indicate vascular involvement (vasculitis, micro thrombo-embolism) in COVID-19 (Figures 11a-11d). Reverse halo sign was mostly seen in patient with organizing pneumonia pattern of injury while halo sign was seen in usual pneumonia pattern. Air-bronchogram was present in cases with consolidations. Reticular sign was associate with either severe involvement or crazy paving.

## Discussion

The previously unknown pathogen in humans is the cause of novel coronavirus pneumonia, which is a severe acute respiratory syndrome. It is characterized by the symptoms of fever, cough, cold, fatigue, anosmia, etc., at the start of the infection and is also highly contagious in nature. The severe cases may present with dyspnea, respiratory distress, and septic shock and may result in death [9].

In the present study, it was found that 50% of the study participants are from the age group of 25-50 years. Similar findings were found in a study done in Maharashtra, India; 48% of the cases were from the adult age group of 20-40 years [10]. In the same study, only 47.3% of cases had an abnormal CT scan, while in the present study, 97% of participants had an abnormal CT scan. In a study of Wuhan, in symptomatic individuals, chest CT scans were frequently abnormal; among 1,014 patients, the chest CT was abnormal in 88% of COVID-19 patients at presentation [11].

The bilateral presence of patchy GGOs is the hallmark of COVID-19. Later, the GGOs may coalesce into dense, consolidative lesions, with a predominantly peripheral distribution under the pleura and along the broncho-vascular bundles [12,13]. In the present study, 97.39% of patients have GGO. In a study in Brazil done during the initial days of a pandemic on 12 COVID-19 patients, 100% of the patients had GGOs [14]. Multi-lobar lung involvement is typical, and the right lower lobe is the most commonly affected lobe [15,16].

In a recent study from Wuhan, about 60%-70% of the patients demonstrated pure GGO on CT [17]. In another study from Wuhan, the most common CT finding was GGO alone. Consolidation was the second most common feature seen in the first 11 days. When combined, GGO and consolidation constituted about 83%-85% of all CT findings in total in the early stage of the disease [18].

CT scans showed a range of features including GGOs, interstitial infiltration, crazy-paving pattern, and multiple patchy consolidations in both lung fields; in addition, vessel enlargement, thick interlobar septa, and air bronchograms were observed [19]. Similar to the present study, in a systematic review and meta-analysis of results from published studies on the detection of COVID-19 by chest CT and the expected CT imaging manifestations of patients who had bilateral lung infection, the incidence was 78.2%, and the lesions were mostly located in the peripheral area [20].

Though COVID-19 infection can involve all lobes, the most commonly involved lobes were the right lower lobe and left lower lobe; 89.56% and 85.21%, respectively. Similarly, Bao et al. found that the right lower lobe and left lower lobe were the most commonly involved; 87.21% and 81.41%. The right upper lobe and left upper lobe were also commonly involved: 65.22% and 69.43%, respectively. In another study, the lower lobes were more inclined to be involved, with higher CT scores [21]. As in the present study, Zhu et al. found that pure pulmonary consolidation seldom, possibly due to the fact that the pure GGO is mostly present in the early stage of the disease [22].

There are some limitations to this study. Clinically more severely affected patients were more likely to undergo HRCT chest which may account for selection bias. Also, some patients might have received medical treatment before being admitted to this institute. This may affect the chest CT finding in these patients and this is not accounted for in our study.

## Conclusions

This article aims to analyze the various radiological patterns observed in COVID-19 lung injuries, with a focus on GGOs, consolidation, crazy paving patterns, and interlobular septal thickening. GGOs and crazy paving patterns were the most common findings.
